# Development of Long-Term Stability of Enveloped rVSV Viral Vector Expressing SARS-CoV-2 Antigen Using a DOE-Guided Approach

**DOI:** 10.3390/vaccines12111240

**Published:** 2024-10-30

**Authors:** MD Faizul Hussain Khan, Caroline E. Wagner, Amine A. Kamen

**Affiliations:** 1 Viral Vectors and Vaccines Bioprocessing Group, Department of Bioengineering, McGill University, Montreal, QC H3A 0E9, Canada; md.f.khan@mail.mcgill.ca; 2 Department of Bioengineering, McGill University, Montreal, QC H3A 0E9, Canada; caroline.wagner@mcgill.ca

**Keywords:** vesicular stomatitis virus (VSV), liquid formulation, viral vaccine bioprocess, enveloped viral vector, stability, pandemics, vaccines, DOE, rVSV-SARS-CoV-2, COVID-19

## Abstract

Liquid formulations have been successfully used in many viral vector vaccines including influenza (Flu), hepatitis B, polio (IPV), Ebola, and COVID-19 vaccines. The main advantage of liquid formulations over lyophilized formulations is that they are cost-effective, as well as easier to manufacture and distribute. However, studies have shown that the liquid formulations of enveloped viral vector vaccines are not stable over extended periods of time. In this study, we explored the development of the liquid formulations of an enveloped recombinant Vesicular Stomatitis Virus (VSV) expressing the SARS-CoV-2 spike glycoprotein. To do so, we used a design of experiments (DOE) method, which allowed us to assess the stability dynamics of the viral vector in an effective manner. An initial stability study showed that trehalose, gelatin, and histidine were effective at maintaining functional viral titers during freeze–thaw stress and at different temperatures (−20, 4, 20, and 37 °C). These preliminary data helped to identify critical factors for the subsequent implementation of the DOE method that incorporated a stress condition at 37 °C. We used the DOE results to identify the optimal liquid formulations under the selected accelerated stress conditions, which then guided the identification of long-term storage conditions for further evaluation. In the long-term stability study, we identified several liquid formulations made of sugar (sucrose, trehalose, and sorbitol), gelatin, and a histidine buffer that resulted in the improved stability of rVSV-SARS-CoV-2 at 4 °C for six months. This study highlights an effective approach for the development of liquid formulations for viral vector vaccines, contributing significantly to the existing knowledge on enveloped viral vector thermostability.

## 1. Introduction

Viral vector vaccines have been shown to induce robust immune responses in several successful COVID-19 vaccines from AstraZeneca/Oxford, Johnson & Johnson, and Russia’s Sputnik V [1]. Replication-deficient recombinant viral vectors are derived from naturally occurring viruses and serve as efficient delivery vehicles for specific genes into target cells. In recent years, improvements in vector design, processing, manufacturing, delivery, and demonstrated safety have resulted in broader applications. This platform holds immense promise in the areas of gene and cell therapy, vaccinations, and cancer treatments [2]. Recombinant Vesicular Stomatitis Virus (VSV) is a preferred viral vector for the development of vaccine candidates due to its stability, adaptability, and capacity to express viral antigens [3,4,5,6]. Genetically modified VSVs that carry foreign antigens have proven to be highly efficient as vector vaccines against various diseases like Ebola and COVID-19. This technology replaces the VSV envelope glycoprotein G with a specific gene of interest, such as the Ebola virus glycoprotein or the SARS-CoV-2 spike protein [7,8].

However, one of the major drawbacks of live viral vector vaccines is their relatively reduced thermostability compared to inactivated or subunit vaccines. Thermostability refers to the ability of a vaccine to maintain its potency when exposed to elevated temperature during storage, transport, and distribution. For instance, the recently approved VSV-based Ebola vaccine, ERVEBO^®^, requires an ultra-low temperature (−70 ± 10 °C) for its whole transportation and storage period. This vaccine is not stable at the conventional refrigerated temperature (4 °C) and can be stored for a maximum of only two weeks under these conditions [9]. This limitation can pose significant challenges, especially in regions with limited infrastructure for cold chain storage and transportation. Maintaining the required cold chain for these vaccines can be logistically complex and costly, whereas deviation from the recommended storage conditions can lead to a reduced efficacy and wastage of vaccine lots.

Compared to non-enveloped viral vectors, enveloped viral vectors exhibit inherent weaknesses due to the presence of an outer lipid membrane. The VSV outer envelope is found to be more rigid, possibly due to high cholesterol concentrations in the viral membrane [5,6,10]. Indeed, cholesterol reduction from the outer membrane of the VSV through the formation of complexes with sphingomyelin and dipalmitoyl lecithin results in a significant loss of viral infectivity, whereas reincorporation of this material into the depleted viral membrane improves infectivity [11,12]. Overall, the integrity of the viral membrane is highly sensitive to both physical and chemical degradation [13,14,15,16]. Chemical degradation can occur through the alteration of covalent bonds via processes such as oxidation and deamidation. On the other hand, physical degradation refers to the aggregation or unfolding of the viral envelope, or its adsorption onto surfaces [17,18]. In extreme conditions, such as elevated temperatures, the viral outer envelope is ruptured, resulting in the inactivation of the virus.

Liquid formulations are used to preserve the structural integrity of viral vectors by preventing denaturation and degradation. They contain protective excipients like stabilizers, surfactants, and osmolytes to protect viruses during storage and transportation [19]. This formulation modality reduces dependency on ultra-low temperature requirements, contributing to improving vaccine access and availability, especially in resource-limited settings. Compared to freeze-drying, another common storage condition under liquid formulations is simpler and more cost-effective [20]. Additionally, liquid formulations may be more stable in some cases than solid formulations that involve encountering stressful conditions on the viral vector during the freeze-drying process.

Excipients like sucrose, trehalose, and gelatin stabilize the enveloped viral particles in liquid formulations through several mechanisms [21]. First, they regulate osmotic pressure, which is crucial for maintaining the structural integrity of viral vectors and viral vaccine components. They also offer protection against chemical degradation by reducing the reactivity of the functional groups within the vaccine components such as phosphate groups (-PO4), and hydroxyl groups (-OH). Sugars can also stabilize the three-dimensional (3D) structure of the viral vector by forming hydrogen bonds with enveloped proteins [13,22], while gelatin enhances viral stability by forming electrostatic interactions, which limits the degradation of surface proteins and lipids. Gelatin can also contribute to the stability of enveloped viruses by providing a surrounding structural matrix, maintaining the virus particles in their native functional state, preventing phase transition, and restricting viral aggregation [21,23]. In Table 1, the optimized liquid formulations of the selected enveloped viral vector vaccines are summarized.

An important challenge in the development of liquid formulations is the difficulty of predicting the long-term stability of viral vectors and viral vaccines [2]. The Design of Experiments (DOE) approach is a method for increasing predictability without requiring an extensive study of a large parameter space of formulations. In this approach, a set of initial experiments is conducted under one or more stress conditions that simultaneously analyze several factors of the vaccine formulation. From these initial experiments, the effect of key formulation parameters such as pH, ionic strength, product concentration, buffers, and stabilizers are identified using statistical analysis techniques such as an analysis of variance (ANOVA), a regression analysis, and the response surface methodology (RSM). By identifying an optimized space of the relevant formulation parameters through the DOE approach, a subsequent set of targeted experiments related to the assessment of the long-term stability of the viral vaccine can be performed.

In this study, we applied the DOE methodology for the development of liquid formulations for an rVSV-SARS-CoV-2 viral vector candidate vaccine. The goal of this work was to identify the formulations and conditions that would enhance the stability of the vector for six months. A preliminary stability study was performed using freeze–thaw stress cycles and short-term exposures at different temperatures. Following the preliminary stability study and DOE analyses, a subsequent set of experiments was conducted under an accelerated stress condition for a short period. Finally, the most effective formulations were assessed in a long-term stability study under various conditions. Overall, the outcomes of this experimental research contribute to improving our capacity to identify potential factors as critical process parameters and provide insights into the possible mechanisms involved in enhancing stability, a critical quality attribute of enveloped viral vectors.

## 2. Materials and Methods

### 2.1. Cell Culture and Virus Infection

This study utilized suspension Vero cells obtained from the National Research Council of Canada for cell culture and viral vector production [28]. These cells were selected due to their potential for scale-up and ability to produce high titers of virus [28]. The rVSV viral construct was obtained from Kang’s laboratory at the University of Western Ontario, in Canada [29,30]. In order to perform both the suspension of Vero cell growth and infection, we used the MDXK medium (Xell AG, Bielefeld, Germany). For the viral infectivity assay, adherent Vero cells were used and cultivated in serum-free VP-SFM (Gibco, Grand Island, NY, USA) [8].

### 2.2. Preparation of Materials

To produce the virus stock for the stability study, Vero cells were grown as a suspension culture in a shake flask at 37 °C and pH 7.2. Media exchange was performed at a targeted cell density ranging between 1 and 2 million/mL and the temperature was reset to 31 °C [30]. The culture was subsequently infected with the virus at a multiplicity of infection (MOI) of 0.01, and cell viability was determined at regular time intervals. The cell viability was found to be approximately 50% at 48 h post-infection (hpi), indicating an effective infection. Then, the harvest was collected and purified through a series of downstream processing steps including benzonase treatment, centrifugation, and anion exchange chromatography [31]. The purified material was subsequently stored at an ultra-low temperature (−80 °C).

### 2.3. Excipients & Buffers

The excipients used for this study were purchased from different sources: sorbitol, trehalose, dextran, and PBS were purchased from Thermo Fisher Scientific, Waltham, MA, USA; sucrose and gelatin (hydrolyzed, 40,000–50,000 Da) from Millipore-Sigma, Burlington, MA, USA; and histidine from VWR, Radnor, Pennsylvania, USA. The excipient formulations used in the various experiments in this study are outlined in Table 2 and Table 3. To prepare the liquid formulation, a total of 1 mL of a solution was prepared in a 1.5 mL microcentrifuge tube where 100 μL of the virus was added to a 900 μL solution containing the excipients. The prepared solutions were filtered through 0.22 μm capsule filters and the tubes were autoclaved before use to prevent unwanted contamination.

### 2.4. DOE Experimental Runs

For the DOE-based approach, the JMP statistical application was used where four design variables were considered within specific ranges to run the DOE (see Table 4). Using these variables, the Box–Behnken design was used to generate response surfaces for visualizing the effect of each variable and their interactions on the infectivity of rVSV-SARS-CoV-2, a critical quality attribute of the viral vaccine product. These 3D response surface plots consist of two independent variables (factors) on the X and Y axes and the dependent variable (the response or infectivity) on the Z axis. We shaded the lower infectivity zones with blue–green color schemes and the higher infectivity zones with red color schemes. The flat surfaces indicate no interactions between the factors, while the curved surfaces indicate significant interactions. The results of the DOE were processed using the JMP application to generate a prediction profiler. This projected the infectivity of rVSV-SARS-CoV-2 taking into account all four parameters across the specified ranges. In this way, the optimal combination of factors for maintaining the infectivity of rVSV-SARS-CoV-2 could theoretically be identified.

The solutions for these experiments were prepared according to the DOE-identified formulations (Supplementary Table S1). Following preparation, the solutions were filtered, the virus suspensions were added, and the experimental runs were conducted at 37 °C for 3 days. The formulations were then assayed using TCID_50_ and the results of the DOE were used by the JMP software version 17.2.0 to construct the effect summary, response surface plots, and prediction profiler.

### 2.5. Infectivity Assay

Tissue culture infectious dose (TCID_50_) assays were used to measure the infectious viral titers. This is a cell culture-based assay using adherent Vero cells for infection. To perform the assay, the cells were quantified using a VI-CELL XR cell counter (Beckman Coulter, Brea, CA, USA) and 100 μL of media containing approximately 15,000 Vero cells were inoculated in each well of a 96-well plate. Adherent Vero cells were grown and maintained in the 96-well plates for 24 h at 37 °C. After 24 h, the used media was replaced with the same volume of fresh media containing a serial dilution of each formulation sample. The plates were kept for four days at 31 °C in a humidified condition containing 5% CO_2_. An inverted microscope was then used to determine viral titer by calculating cytopathogenicity using the Spearman and Kärber algorithm (mean coefficient of variation: 23.6%; Supplementary Figure S1) [8,32]. Cytopathogenicity refers to the visible changes that occur in cells as a result of a viral infection. VSV-induced CPE is characterized by changing the morphology of the Vero cell from a typical flat shape to a more rounded one. After determining the viral titer, the loss of viral titers was expressed as the log loss infectivity defined as the following:

Log loss = Log titer (−80 °C stock) − Log titer (formulation sample)

### 2.6. Modeling & Statistical Analysis

To compare the formulated and unformulated (control) groups, the one-way analyses of variance (ANOVA) by GraphPad Prism version 9.4.1 was used. The data in this investigation followed a Gaussian distribution, as verified by quantile–quantile (QQ) plots. Dunnett’s multiple comparison tests were conducted at a 95% confidence interval of difference. The statistically significant differences between these groups were identified at a *p*-value below 0.05 and were denoted by asterisks (*). The JMP application was used to construct the DOE-based response surface design plots and subsequent effect summary and prediction profiler.

## 3. Results

### 3.1. Preliminary Stability Study

**Figure 1:**
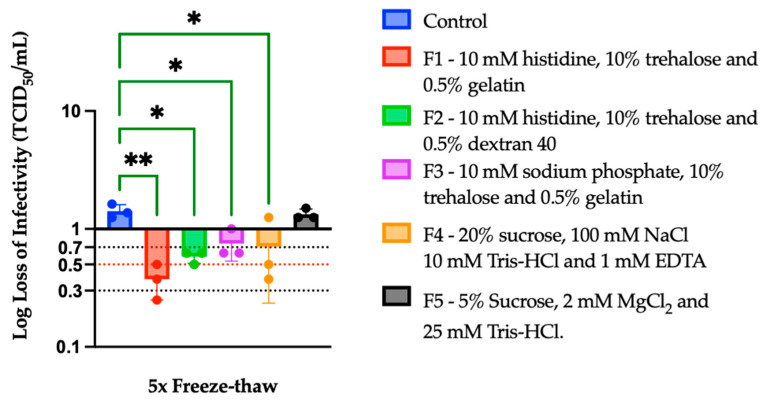
Preliminary stability analysis of exposure of rVSV-SARS-CoV-2 to five cycles of freeze–thaw stress. A TCID_50_ infectivity assay was used to determine the functional viral titer in all samples (*n* = 3). Significant statistical differences between the control and test formulations were determined by one-way ANOVA analysis and are denoted through the asterisk (*) sign. Statistically significant differences were found at *p* < 0.05 (** *p* < 0.01; * *p* < 0.05).

A preliminary stability study was carried out by applying two independent stress conditions: freeze–thaw stress and temperature stress. Five formulations were initially developed to determine their stabilizing efficacy (see Table 2). Freeze–thaw stress cycles were applied to all the formulations by freezing them at −80 °C and immediately thawing them at 37 °C for five cycles. Here, the maximum recovery of the infectious viral titer (~0.4 log loss of infectivity, the log loss formula is presented in Section 2.5 of the Methods Section) was observed in the F1 formulation, which contained histidine, trehalose, and gelatin. The ANOVA revealed comparatively higher statistically significant differences between the control and F1, suggesting that F1 significantly reduced the loss of rVSV-SARS-CoV-2 infectious viral titers. Conversely, the F5 formulation (5% sucrose) and the control showed the lowest recovery, each exhibiting a loss of infectious viral titers of approximately 2 to 3 logs. Here, no statistically significant difference was found between the control and F5, showing the inefficacy of the F5 formulation. The other three formulations F2, F3, and F4 were also effective at reducing the loss of infectious viral titers, although to a lesser degree than F1 (Figure 1).

**Figure 2:**
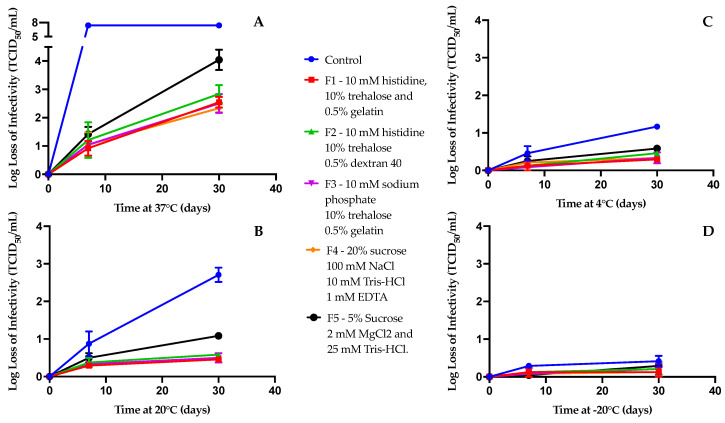
Stability analysis of five distinct formulations of rVSV-SARS-CoV-2 at different temperatures of 37 °C (**A**), 20 °C (**B**), 4 °C (**C**), and −20 °C (**D**) at one week and one month. A TCID_50_ infectivity assay was used to determine the functional viral titer in all samples (*n* = 3). Here, the viral titer is presented as a log loss of infectivity on the y axis.

Additionally, to observe their efficacy in stabilizing the viral vector, these five formulations were kept at four different temperatures (−20, 4, 20, and 37 °C) for one week and one month. Of the four different temperatures, the highest stress was observed at 37 °C, where all the formulations showed a significant loss of infectivity (>2 log loss) within a short time (Figure 2A) and no infectious viral titer was detected after one week in the control (unformulated) experiments. At this high-stress temperature, the formulations with high sucrose concentrations (F4, 20% sucrose) and 10% trehalose with 0.5% gelatin (F2 and F3) were the most effective at preserving the infectivity. These formulations also showed an enhanced stability at the other temperatures tested (−20, 4, and −20 °C) over one month (Figure 2B–D). The F5 formulation (5% sucrose) showed a substantial loss of infectious viral titers, particularly at 20 and 37 °C.

Overall, we found that the decline in the infectious viral titer was much less at 20 °C than at 37 °C (compare Figure 2A and Figure 2B). At 20 °C, formulations F1 to F4 showed a relatively high stability (0.3 to 0.5 log loss of infectivity) after one month. Moreover, further reducing the temperature led to a noticeable increase in stability. At 4 °C and −20 °C (Figure 2C,D), all the formulations showed ≲0.5 log losses of infectivity.

### 3.2. DOE-Based Accelerated Stress Study

**Figure 3:**
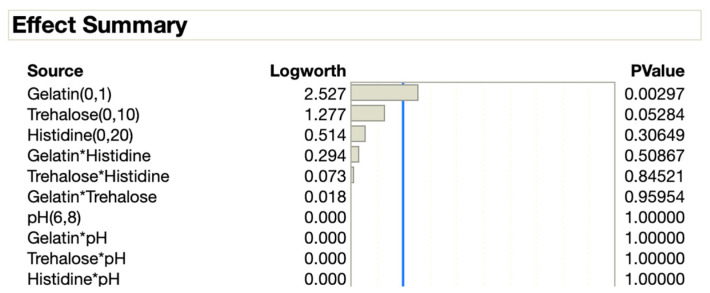
The effect summary shows the standardized effects of several factors on rVSV-SARS-CoV-2 stability. The effect summary was created by the JMP application using the *p*-value generated from the DOE-based experimental results. Here, the blue line indicates the significance boundary (*p* < 0.05).

From the preliminary stability study in Section 3.1, four design variables were selected for the DOE approach (Table 4), and all the DOE formulations were kept at the accelerated stress condition of 37 °C for three days. For these experimental runs, 100 μL of purified virus solution was added to 900 μL of each of the formulations (Supplementary Table S1). The results of these experiments were fed into the JMP application, resulting in the effect summary shown in Figure 3. A low *p*-value in this study signifies that the findings are reproducible and suggests that the factor has a significant impact. Based on the effect summary, it is noted that gelatin has the most significant impact on the stability of rVSV-SARS-CoV-2 among all the considered variables. The second most important factor was found to be trehalose followed by histidine, gelatin*histidine, and so on (Figure 3). Here, gelatin*histidine means these two factors were analyzed together, and as such it captures their interaction. In other words, it considers how the combined presence of gelatin and histidine affected viral stability rather than each component individually.

**Figure 4:**
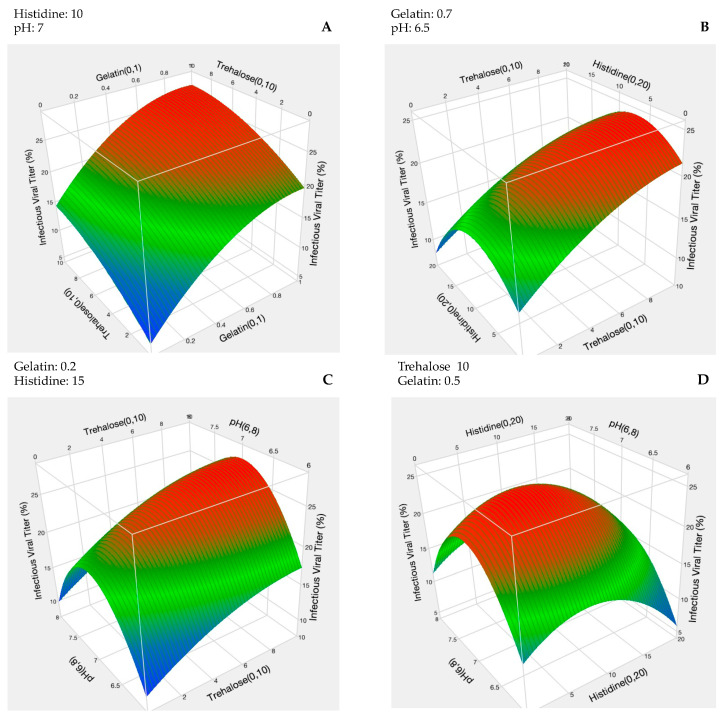
Response surface plots between two individual variables from the DOE accelerated stress study and the infectivity of rVSV_SARS-CoV-2 including the (**A**) gelatin–trehalose interaction, (**B**) trehalose–histidine interaction, (**C**) trehalose–pH interaction, and (**D**) histidine–pH interaction. The units of trehalose and gelatin are in percentages and histidine is in mM.

The result of the DOE study was also used to develop the 3D response surface plots shown in Figure 4. These 3D plots serve to visualize the interactions between two given factors, and their impact on the infectivity of rVSV-SARS-CoV-2. In Figure 4A, the response surface plot for gelatin and trehalose is shown, and virus infectivity is shown to increase monotonically with the increasing concentrations of both compounds. The interaction of trehalose with histidine (Figure 4B) and trehalose with pH (Figure 4C) again showed that viral infectivity increased with a higher trehalose concentration. However, viral infectivity was maximized at moderate pH and histidine levels. Finally, in Figure 4D where the interaction between histidine and pH is shown, the red zone of high viral infectivity coincides with a moderate pH (pH approximately 7.00) and moderate histidine concentrations (approximately 10 mM).

**Figure 5:**
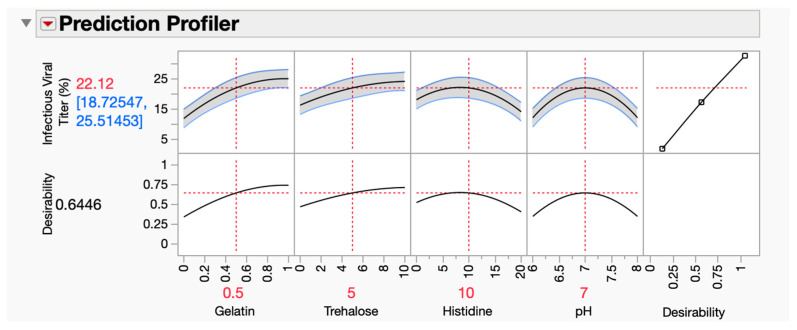
The prediction profiler was developed by the JMP application using the design of experiments (DOE) output. A total of four variables were used by the prediction profiler: trehalose, gelatin, pH, and a histidine buffer. Here, changing the input of the variables gives the final output as a percentage of rVSV-SARS-CoV-2 infectivity.

The results of the DOE were processed using the JMP application to generate a prediction profiler (Figure 5). This projected the infectivity of rVSV-SARS-CoV-2 by taking into account all four parameters across the specified ranges. Consistent with the response surface plots, viral infectivity increases uniformly with the increasing concentrations of gelatin and trehalose, and the highest viral infectivity is found at the highest concentrations tested (10% for trehalose and 1% for gelatin). Conversely, the histidine and pH curves show the lowest viral infectivity at both endpoints, peaking at the mid-region of approximately 10 mM of histidine and a pH of 7.00.

### 3.3. Long-Term Stability Study

The main objective of the implementation of the DOE in this study was to predict the optimized formulations that will be effective in stabilizing rVSV-SARS-CoV-2 during long-term storage. As a result, in our final set of experiments, we performed a long-term stability study at 4 and 20 °C for one and six months with trehalose containing four formulations derived from the DOE experiments as well as a control formulation (see Table 3).

**Figure 6:**
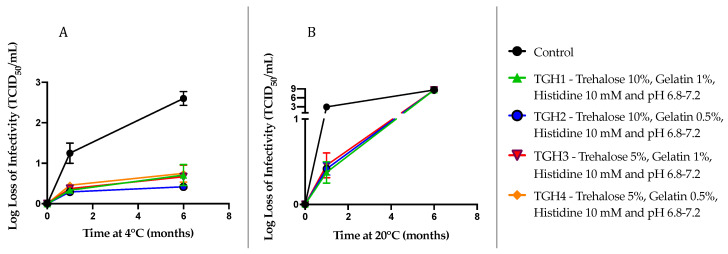
Long-term stability data for four distinct formulations of rVSV-SARS-CoV-2 at 4 °C (**A**) and 20 °C (**B**). A TCID_50_ infectivity assay was used to determine the functional viral titer in all samples (*n* = 3). Here, the viral titer is presented as a log loss of infectivity on the y axis.

All formulations showed a significant loss of the infectious viral titer of rVSV-SARS-CoV-2 after six months at 20 °C (Figure 6B). After one month, there were no significant differences in the stability of rVSV-SARS-CoV-2 at both temperature conditions for all the formulations. However, after six months the differences were substantial. At 4 °C, the log loss infectivity of rVSV-SARS-CoV-2 for all the formulations fell below 1 log loss, indicating a high stability (Figure 6A). In contrast, the control group exhibited around a 2.5 log loss of the infectious viral titer. Among these formulations, TGH2 (10% trehalose, 0.5% gelatin, and 10 mM histidine) showed an improved stability with a log loss of 0.4 ± 0.1. TGH1 (10% trehalose, 1% gelatin, and 10 mM histidine) showed a 0.7 to 1 log loss of infectivity, which was close to the other two formulations TGH3 (5% trehalose, 1% gelatin, and 10 mM histidine) and TGH4 (5% trehalose, 0.5% gelatin, and 10 mM histidine). Based on the results of the DOE, we had anticipated that TGH1 would be the formulation with the greatest long-term stability. In contrast, we found that the TGH2 formulation with a lower gelatin concentration (0.5%) remained the most stable after 6 months at 4 °C.

**Figure 7:**
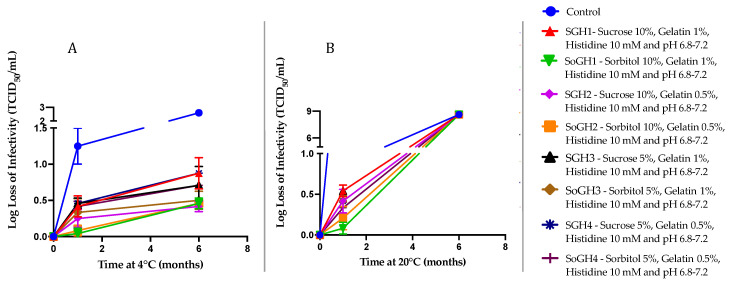
Long-term stability data for eight distinct formulations of rVSV-SARS-CoV-2 at 4 °C (**A**) and 20 °C (**B**). A TCID_50_ infectivity assay was used to determine the functional viral titer in all samples (*n* = 3). Here, the viral titer is presented as a log loss of infectivity on the y axis.

As a final step, we extrapolated the DOE results by replacing trehalose with two similar types of sugars, sucrose and sorbitol. To do so, we added sucrose and sorbitol in mixtures and proportions identical to those used with trehalose (see Table 3), creating eight additional formulations. Similar to TGH2, after six months at 4 °C, SGH2 (10% sucrose, 0.5% gelatin, and 10 mM histidine) and SoGH2 (10% sorbitol, 0.5% gelatin, and 10 mM histidine) showed improved stability (~0.4 log loss of infectivity, Figure 7A). Also, the sucrose-containing SGH1 with a high gelatin concentration (1%) showed a 0.7 to 1 log loss of infectivity under these conditions, while the sorbitol-containing SoGH1 with 1% gelatin demonstrated an enhanced stability (~0.5 log loss of infectivity). Consistent with the trehalose results, after six months at 20 °C (Figure 7B), all the formulations showed a complete loss of infectious viral titers.

## 4. Discussion

The development of engineered viral vector vaccines against various viral diseases and cancer is gaining attention. However, vaccine stability during storage, transport, and administration is a major challenge affecting equitable access to vaccines. To overcome this limitation, stable and suitable liquid formulations for viral vector vaccines are needed. [32]. This manuscript focuses on the development and characterization of a liquid formulation for the enveloped viral vector rVSV-SARS-CoV-2, which can enhance the vaccine’s shelf life, simplify transportation and storage, and improve delivery and administration to patients.

A wide variety of excipients and physiological conditions have been exploited to enhance the formulation of various viral vector vaccines and similar biopharmaceutical compounds. The findings from our preliminary stability study showed that trehalose combined with gelatin can contribute to retaining the infectivity of rVSV-SARS-CoV-2 in freeze–thaw and temperature-stress conditions. Also, increased concentrations of sucrose reduced the infectious VSV viral titer loss by a significant margin. Previous studies also showed that sucrose and trehalose maintain the monomeric state of the proteins within the vaccine by preventing the formation of aggregates [19,24]. In terms of a mechanism, sugars can maintain pH-stable environments by functioning as pH buffers. Here, pH stability is critical for preserving the structural and functional integrity of the viral vector. In the case of recombinant human cytomegalovirus, 10% of sucrose, sorbitol, and trehalose were identified as effective stabilizers [24]. Additionally, gelatin has been utilized for decades in the formulation of vaccines against influenza, rubella, shingles, zoster, varicella, measles, mumps, and more [21].

The preliminary stability study helped to identify the critical variables and stress conditions to implement in the DOE set-up. Here, four variables were selected for the DOE approach including the excipients and the physiological condition (pH). Among all the variables, gelatin showed the highest impact on the stability of the vector followed by trehalose and histidine concentration. It has been found that viral titer is dramatically reduced at extreme pH ranges, especially below a pH of 6 and above a pH of 8 [24]. The buffer also played a vital role in the stability of the viral vector, and optimum buffer concentrations are crucial to maintaining viruses in viable states. For instance, for cytomegalovirus, histidine buffers have shown improved stability compared to sodium phosphate and Tris [24]. Here, the optimal range of histidine buffers for viral vectors was determined to be between 0 and 20 mM.

In the DOE-based formulation development, an accelerated stress study is selected that mimics the extreme conditions a product may encounter over an extended period. This is performed to optimize the formulations, extend product shelf life, and ensure consistent quality under various environmental conditions [33,34]. Here, the stress condition (37 °C) was selected based on the observation during the preliminary stability study that it produced an extreme loss of viral titers. From the DOE, the prediction profiler developed a model forecasting the vector’s infectivity within the specific variable ranges. It hypothesized that the most favorable formulations would have high concentrations of sugar and gelatin with 10 mM histidine and an optimal pH of 7.00.

In terms of long-term stability, we found that a high concentration of sugar and a medium concentration of gelatin promoted the highest viral infectivity at 4 °C (SGH2, TGH2, and SoGH2). One possible explanation for why high gelatin concentrations were more effective during the preliminary stability experiments and the DOE is that a higher concentration of gelatin (1%) was in a liquid state at a high temperature (37 °C), whereas it is in a solid state at refrigerated temperatures (4 °C), This suggests that gelatin may work better as a stabilizing agent in its liquid state with 0.5% gelatin at 4 °C in the long term. Also, the optimized range of pH (~7.00) and histidine (10 mM) combined with the other two parameters showed improved stability. Importantly, this study identified four stable liquid formulations for rVSV-SARS-CoV-2 for long-term stability (SoGH1, TGH2, SGH2, and SoGH2). Overall, this study highlights the significance of a systematic approach for the development of liquid formulations, incorporating prior knowledge of the relevant literature and the implementation of a DOE approach.

Despite the success in developing stable formulations, this study is not without limitations. This DOE-based stability study did not cover a wide range of the excipients and factors that might influence the stability of the enveloped viral vector vaccine. Additionally, selecting the stress conditions for DOE-based experiments presents a challenge as different factors might affect the stability of the enveloped viral vector vaccine differently under various storage conditions. Therefore, building on the findings of the current study, further experiments incorporating different excipients and stress conditions are needed to establish the ideal formulation conditions for thermostable VSV-vectored vaccines.

Several vaccines are under development using the rVSV platform, which has shown promise in clinical trials against Ebola, Marburg, Lassa, Nipah, Zika, CCHF, MERS, and SARS [8]. The International AIDS Vaccine Initiative (IAVI)’s Lassa Virus (LASV) vaccine candidate uses the same rVSV vector platform. The phase I LASV vaccine trial demonstrated robust immune responses that lasted up to a year post-vaccination, and currently, the vaccine is undergoing a phase 2 clinical trial [35]. The recently licensed VSV-based Ebola vaccine Ervebo, however, necessitates storage temperatures below −60 °C. After thawing, the vaccine can only be stored at refrigerated temperatures (2–8 °C) for up to 2 weeks [9]. The outcome of the present study provides some potential stable liquid VSV formulations to address these instability challenges. These liquid formulations of VSV are considered easier to use, cost-effective, and offer more flexible options for vaccine transport and delivery. By improving the long-term stability of the liquid formulations of VSV-based rVSV-SARS-CoV-2 vaccines across a range of temperatures, we demonstrate the potential to distribute effective vaccines more efficiently globally. The optimized liquid formulations identified from this study have achieved long-term stability at 2–8 °C and can be manufactured at a low cost on a large industrial scale.

## 5. Conclusions

This study demonstrates that the combination of a DOE-based liquid formulation design with appropriate accelerated stress condition experiments supports the effective prediction of the critical parameters determining the long-term stability of a vaccine candidate. Using this approach, we successfully developed a simplified liquid formulation with sucrose, trehalose, and sorbitol which contributed to significantly enhance the stability of a VSV candidate vaccine. The addition of sugars in the formulation reduce heat-induced denaturation and prevent aggregation of VSV particles by stabilizing viral capsid proteins. Additional studies can certainly expand on multiple dimensions of the formulation operating space to further improve the prediction for long term viral vector stability. To validate such protocols and increase their generalization and acceptance, the stability of other viral vectored candidate vaccines should also be tested using the identified formulations to increase and eventually broaden the database. Ultimately, a systematic and quantitative approach to comprehending the mechanisms associated with the thermostability of viral vectors might contribute to increasing the prediction capacity and to significantly reducing the need of repeated, costly, and time-consuming one factor at a time formulation experiments traditionally guided only by empirical approaches.

## Figures and Tables

**Table 1:** Liquid formulations developed for selected enveloped viral vaccines.

Virus	Type	Composition	Ref.
Human Cytomegalovirus vector (rHCMV-1)	DNA virus.	Trehalose, sucrose, sorbitol, and hydrolyzed gelatin, dextran 40, pH 7.00	[24]
Influenza virus	RNA viruses.	Sucrose (2.5%), arginine (1%), and human serum albumin (0.5%), optimum pH 6.5–6.7	[25]
Bovine herpesvirus (BHV)	DNA virus.	5% hydrolyzed gelatin	[20]
Classical swine fever virus (CSF)	RNA viruses.	Lactalbumin hydrolysate-Trehalose	[26]
Flavivirus	RNA viruses.	Trehalose, albumin and a pluronic polymer	[27]

**Table 2:** Excipient formulations for preliminary stability study of rVSV-SARS-CoV-2.

Control	F1	F2	F3	F4	F5
Neither excipient nor buffer was added. Only Mili Q water.	10 mM histidine	10 mM histidine	10 mM sodium phosphate	20% sucrose	5% sucrose
	10% trehalose	10% trehalose	10% trehalose	100 mM NaCl	2 mM MgCl_2_ and
	0.5% gelatin	0.5% dextran 40	0.5% gelatin	10 mM Tris-HCl	25 mM Tris-HCl.
				1 mM EDTA	
	pH 7.05	pH 6.94	pH 6.87	pH 7.14	pH 6.92

**Table 3:** Excipient formulations for long-term stability study of rVSV-SARS-CoV-2.

TGH1		TGH2		TGH3		TGH4	
Trehalose	10%	Trehalose	10%	Trehalose	5%	Trehalose	5%
Gelatin	1%	Gelatin	0.5%	Gelatin	1%	Gelatin	0.5%
Histidine	10 mM	Histidine	10 mM	Histidine	10 mM	Histidine	10 mM
pH	6.8–7.2	pH	6.8–7.2	pH	6.8–7.2	pH	6.8–7.2
SGH1		SGH2		SGH3		SGH4	
Sucrose	10%	Sucrose	10%	Sucrose	5%	Sucrose	5%
Gelatin	1%	Gelatin	0.5%	Gelatin	1%	Gelatin	0.5%
Histidine	10 mM	Histidine	10 mM	Histidine	10 mM	Histidine	10 mM
pH	6.8–7.2	pH	6.8–7.2	pH	6.8–7.2	pH	6.8–7.2
SoGH1		SoGH2		SoGH3		SoGH4	
Sorbitol	10%	Sorbitol	10%	Sorbitol	5%	Sorbitol	5%
Gelatin	1%	Gelatin	0.5%	Gelatin	1%	Gelatin	0.5%
Histidine	10 mM	Histidine	10 mM	Histidine	10 mM	Histidine	10 mM
pH	6.8–7.2	pH	6.8–7.2	pH	6.8–7.2	pH	6.8–7.2

**Table 4:** Selection of design variables for the design of experiments (DOE).

Four Design Variables				
Variables	Min	Mid	Max	Unit
**Gelatin Concentration**	0	0.5	1	%
**Buffer Concentration**	0	10	20	mM
**Trehalose Concentration**	0	5	10	%
**pH**	6	7	8	

## Data Availability

The original contributions presented in this study are included in this article; further inquiries can be directed to the corresponding author.

## Supplementary Materials

The following supporting information can be downloaded at https://www.mdpi.com/article/10.3390/vaccines12111240/s1: Table S1: DOE developed from Response Surface Design by JMP software; Figure S1: To quantify the variability of the TCID_50_ infectivity test, we conducted three replicates of the same material at 0 days and at 1-month intervals.
