# Supplementary material for: Development of Long-Term Stability of Enveloped rVSV Viral Vector Expressing SARS-CoV-2 Antigen Using a DOE-Guided Approach
Source: Vaccines (Basel). 2024 Oct 30;12(11):1240. doi: 10.3390/vaccines12111240 (PMC11598241; doi:10.3390/vaccines12111240)
Supplement: Supplementary file 1 [file vaccines-12-01240-s001.zip › vaccines-3220278-supplementary.pdf]

Supplementary Material

Supplementary Table S1. DOE developed from Response Surface Design by JMP software.

Pattern	Gelatin	Trehalose	Histidine	pH
**−−00**	0	0	10	7
**−0−0**	0	5	0	7
**−00−**	0	5	10	6
**−00+**	0	5	10	8
**−0+0**	0	5	20	7
**−+00**	0	10	10	7
**0−−0**	0.5	0	0	7
**0−0−**	0.5	0	10	6
**0−0+**	0.5	0	10	8
**0−+0**	0.5	0	20	7
**00−−**	0.5	5	0	6
**00−+**	0.5	5	0	8
**0000**	0.5	5	10	7
**0000**	0.5	5	10	7
**0000**	0.5	5	10	7
**00+−**	0.5	5	20	6
**00++**	0.5	5	20	8
**0+−0**	0.5	10	0	7
**0+0−**	0.5	10	10	6
**0+0+**	0.5	10	10	8
**0++0**	0.5	10	20	7
**+−00**	1	0	10	7
**+0−0**	1	5	0	7
**+00−**	1	5	10	6
**+00+**	1	5	10	8
**0000**	1	5	20	7
**0000**	1	10	10	7

A

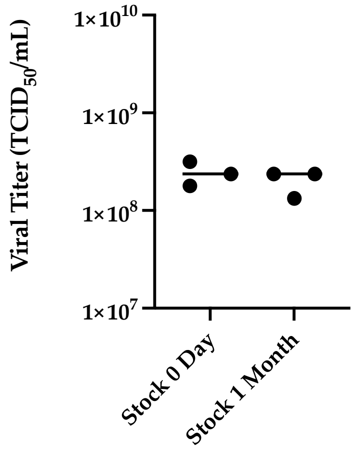

B

	Stock 0 Day	Stock 1 Month
**Mean**	2.44E+08	2.03E+08
**SD**	5.67E+07	4.89E+07
**Coefficient of variability (%)**	23.26069621	24.15536242

Supplementary Figure S1. To quantify the variability of the $TCID_{50}$ infectivity test, we conducted three replicates of the same material at 0 days and 1 month intervals. The mean coefficient of variability between both circumstances was quantified to be 23.6%.
